# Supplementary material for: Opioid prescribing in out-of-hours primary care in Flanders and the Netherlands: A retrospective cross-sectional study
Source: PLoS One. 2022 Apr 7;17(4):e0265283. doi: 10.1371/journal.pone.0265283 (PMC8989290; doi:10.1371/journal.pone.0265283)
Supplement: S1 Table — (DOCX) [file pone.0265283.s001.docx]

**Table S1A. Number of contacts with at least one opioid prescription at the OOH-PCS per 1000 OOH-PCS contacts in 2015-2019 in the Netherlands^#^**

| **ATC** | **Opioid** | 2015 | 2016 | 2017 | 2018 | 2019 |
| --- | --- | --- | --- | --- | --- | --- |
|  | **Total** | 19.76 | 22.14 | 24.45 | 23.37 | 21.41 |
|  | **Weak*** | 10.09 | 10.78 | 11.20 | 10.64 | 9.29 |
| N02BE51 | Codeine and paracetamol | 0.47 | 0.40 | 0.16 | 0.10 | 0.04 |
| N02AA59 |  | 0.24 | 0.20 | 0.10 | 0.07 | 0.03 |
| N02AJ06 |  | - | - | 0.17 | 0.19 | 0.13 |
| N02AX02 | Tramadol | 7.66 | 8.46 | 9.07 | 8.60 | 7.77 |
| N02AJ13 | Tramadol and paracetamol | - | - | 0.68 | 0.74 | 0.88 |
| N02AX52 |  | 1.73 | 1.74 | 1.04 | 0.95 | 0.44 |
|  | **Strong*** | 9.73 | 11.43 | 13.32 | 12.83 | 12.26 |
| N02AA01 | Morphine | 5.32 | 6.26 | 7.11 | 6.85 | 6.88 |
| N02AA51 |  | - | - | - | 0.00 | - |
| N02AA05 | Oxycodone | 3.93 | 4.72 | 5.68 | 5.51 | 4.96 |
| N02AA55 | Oxycodone and naloxone | - | 0.00 | 0.00 | 0.00 | 0.00 |
| N02AB03 | Fentanyl | 0.74 | 0.76 | 0.91 | 0.95 | 0.95 |
| N02AA03 | Hydromorphone | 0.01 | 0.00 | 0.00 | 0.00 | 0.00 |
| N02AX06 | Tapentadol | - | 0.00 | 0.00 | 0.01 | 0.01 |
| N02AE01 | Buprenorphine | 0.08 | 0.10 | 0.11 | 0.09 | 0.08 |

#Numbers include all contacts, both during evenings and nights of weekdays and during weekends.
*Numbers of weak and strong opioid do not add up to the total as both a weak and a strong opioid may have been prescribed during a contact.

**Table S1B. Number of contacts with at least one opioid prescription in the weekend at the OOH-PCS per 1000 OOH-PCS weekend-contacts in 2015-2019 in Flanders and the Netherlands**

| ATC | Opioid | 2015 | | 2016 | | 2017 | | 2018 | | 2019 | |
| --- | --- | --- | --- | --- | --- | --- | --- | --- | --- | --- | --- |
|  |  | FL | NL | FL | NL | FL | NL | FL | NL | FL | NL |
|  | **Total** | 24.13 | 21.70 | 21.44 | 25.33 | 20.57 | 28.03 | 20.93 | 27.60 | 23.46 | 25.94 |
|  | **Weak*** | 21.74 | 11.03 | 19.84 | 12.25 | 19.16 | 12.49 | 19.60 | 11.95 | 21.65 | 10.70 |
| N02BE51 | Codeine and paracetamol | - | 0.65 | - | 0.54 | - | 0.22 | - | 0.13 | - | 0.06 |
| N02AA59 |  | 5.27 | 0.30 | 4.09 | 0.26 | 3.60 | 0.12 | 3.41 | 0.08 | 3.65 | 0.04 |
| N02AJ06 |  | - | - | - | - | - | 0.23 | - | 0.25 | - | 0.19 |
| N02AX02 | Tramadol | 8.23 | 8.14 | 8.39 | 9.32 | 7.94 | 9.84 | 9.28 | 9.36 | 11.41 | 8.83 |
| N02AJ13 | Tramadol and paracetamol | - | - | - | - | 0.23 | 0.83 | 0.98 | 0.93 | 1.78 | 1.06 |
| N02AX52 |  | 8.23 | 1.96 | 7.37 | 2.14 | 7.39 | 1.25 | 5.92 | 1.20 | 4.80 | 0.53 |
|  | **Strong*** | 2.08 | 10.77 | 1.56 | 13.22 | 1.41 | 15.74 | 1.33 | 15.86 | 1.81 | 15.46 |
| N02AA01 | Morphine | 0.89 | 6.69 | 0.50 | 8.20 | 0.46 | 9.41 | 0.41 | 9.55 | 0.91 | 9.77 |
| N02AA51 |  | - | - | - | - | - | - | - | 0.00 | - | - |
| N02AA05 | Oxycodone | 0.59 | 3.91 | 0.48 | 4.85 | 0.51 | 6.13 | 0.47 | 6.22 | 0.56 | 5.71 |
| N02AA55 | Oxycodone and naloxone | 0.02 | - | - | 0.00 | - | - | 0.01 | 0.00 | - | - |
| N02AB03 | Fentanyl | 0.48 | 0.67 | 0.55 | 0.74 | 0.38 | 0.90 | 0.43 | 0.99 | 0.32 | 1.02 |
| N02AA03 | Hydromorphone | - | 0.00 | - | 0.00 | 0.02 | 0.00 | - | - | 0.02 | 0.00 |
| N02AX06 | Tapentadol | - | - | - | - | - | - | - | 0.00 | - | 0.00 |
| N02AE01 | Buprenorphine | 0.10 | 0.06 | 0.02 | 0.08 | 0.02 | 0.10 | 0.01 | 0.07 | 0.01 | 0.07 |

*Numbers of weak and strong opioid do not add up to the total, as both a weak and a strong opioid may have been prescribed during a contact. FL=Flanders, NL=the Netherlands
