# Supplementary material for: Opioid prescribing in out-of-hours primary care in Flanders and the Netherlands: A retrospective cross-sectional study
Source: PLoS One. 2022 Apr 7;17(4):e0265283. doi: 10.1371/journal.pone.0265283 (PMC8989290; doi:10.1371/journal.pone.0265283)
Supplement: S2 Table — (DOCX) [file pone.0265283.s002.docx]

**Table S2: Top 10 diagnoses of OOH contacts with >=1 opioid prescription in Flanders and the Netherlands in 2018**

**Table S2A: Top 10 diagnoses of OOH contacts with at least one strong opioid prescription in Flanders in 2018**

| **ICPC-code** | **ICPC omschrijving** | **%** |
| --- | --- | --- |
| A99 | General disease NOS | 14.5 |
| L02 | Back symptom/complaint | 12.0 |
| P04 | Feeling/behaving irritable/angry | 4.3 |
| R02 | Shortness of breath/dyspnoea | 4.3 |
| R99 | Respiratory disease other | 3.4 |
| A07 | Coma | 2.6 |
| L79 | Sprain/strain of joint NOS | 2.6 |
| L86 | Back syndrome with radiating pain | 2.6 |
| L99 | Musculoskeletal disease, other | 2.6 |
| D82 | Teeth/gum disease | 2.6 |

**Table S2B: Top 10 diagnoses of OOH contacts with at least one weak opioid prescription in Flanders in 2018**

| **ICPC-code** | **ICPC omschrijving** | **%** |
| --- | --- | --- |
| L03 | Low back symptom/complaint [ex. L86] | 15.5 |
| D82 | Teeth/gum disease | 10.0 |
| L86 | Back syndrome with radiating pain | 5.9 |
| L02 | Back symptom/complaint | 5.5 |
| L01 | Neck symptom/compliant [ex. N01] | 4.6 |
| L83 | Neck syndrome | 3.1 |
| R74 | Upper respiratory infection acute | 2.8 |
| L92 | Shoulder syndrome | 2.8 |
| L18 | Spierpijn | 2.2 |
| L79 | Sprain/strain of joint NOS | 2.1 |

**Table S2C: Top 10 diagnoses of OOH contacts with at least one strong opioid prescription in the Netherlands in 2018**

| **ICPC-code** | **ICPC omschrijving** | **%** |
| --- | --- | --- |
| U95 | Urinary calculus | 9.0 |
| D06 | Abdominal pain localized other | 7.7 |
| L86 | Back syndrome with radiating pain | 6.6 |
| A01 | Pain general/multiple sites | 5.1 |
| L02 | Back symptom/complaint | 4.6 |
| D98 | Cholecystitis/cholelithiasis | 3.4 |
| A13 | Concern/fear medical treatment | 2.9 |
| D01 | Abdominal pain/cramps general | 2.7 |
| L03 | Low back symptom/complaint | 2.7 |
| R02 | Shortness of breath/dyspnoea | 2.4 |

** 4.5 % of contacts had missing ICPC codes. These were included for calculating the percentages*

**Table S2D: top 10 diagnoses of OOH contacts with at least one weak opioid prescription in the Netherlands in 2018**

| **ICPC-code** | **ICPC omschrijving** | **%** |
| --- | --- | --- |
| L02 | Back symptom/complaint | 9.3 |
| L86 | Back syndrome with radiating pain | 7.2 |
| L03 | Low back symptom/complaint [ex. L86] | 5.9 |
| L08 | Shoulder symptom/complaint | 4.7 |
| D06 | Abdominal pain localized other | 4.5 |
| L04 | Chest symptom/complaint | 4.4 |
| L81 | Injury musculoskeletal NOS | 3.1 |
| L01 | Neck symptom/compliant [ex. N01] | 2.9 |
| D19 | Teeth/gum symptom/complaint | 2.8 |
| L14 | Leg/thigh symptom/complaint | 2.8 |

** 5.6 % of contacts had missing ICPC codes. These were included for calculating the percentages*
