# Supplementary material for: Opioid prescribing in out-of-hours primary care in Flanders and the Netherlands: A retrospective cross-sectional study
Source: PLoS One. 2022 Apr 7;17(4):e0265283. doi: 10.1371/journal.pone.0265283 (PMC8989290; doi:10.1371/journal.pone.0265283)
Supplement: S3 Table — (DOCX) [file pone.0265283.s003.docx]

**Table S3. Sex and age distribution of patients with an opioid prescription at the OOH-PCS, in Flanders and the Netherlands in 2015-2019**

|  | **Flanders** | | | | | **the Netherlands** | | | | |
| --- | --- | --- | --- | --- | --- | --- | --- | --- | --- | --- |
|  | **2015** | **2016** | **2017** | **2018** | **2019** | **2015** | **2016** | **2017** | **2018** | **2019** |
| **Total** |  |  |  |  |  |  |  |  |  |  |
| Sex (% females) | 59.9 | 54.2 | 57.0 | 57.7 | 58.2 | 57.0 | 57.6 | 57.2 | 56.7 | 56.4 |
| Age category (%) |  |  |  |  |  |  |  |  |  |  |
| 0-14 | 0.5 | 0.1 | 0.2 | 0.2 | 0.2 | 0.1 | 0.2 | 0.2 | 0.1 | 0.1 |
| 15-24 | 5.4 | 6.1 | 5.3 | 6.8 | 6.0 | 5.7 | 5.9 | 5.7 | 5.6 | 5.3 |
| 25-44 | 34.9 | 36.6 | 36.7 | 36.4 | 32.7 | 25.9 | 26.4 | 26.6 | 26.6 | 26.0 |
| 45-64 | 33.8 | 35.5 | 33.4 | 33.8 | 33.6 | 32.4 | 32.7 | 32.2 | 32.2 | 32.4 |
| 65-74 | 9.8 | 7.7 | 10.0 | 11.0 | 9.9 | 13.4 | 13.0 | 13.4 | 13.4 | 13.7 |
| 75+ | 15.7 | 14.1 | 14.3 | 11.9 | 17.6 | 22.5 | 21.9 | 21.9 | 21.9 | 22.4 |
| **Weak opioids** |  |  |  |  |  |  |  |  |  |  |
| Sex (% females) | 58.9 | 59.5 | 57.0 | 58.2 | 58.1 | 58.3 | 59.1 | 58.8 | 58.5 | 58.2 |
| Age category (%) |  |  |  |  |  |  |  |  |  |  |
| 0-14 | 0.5 | 0.1 | 0.3 | 0.2 | 0.2 | 0.2 | 0.2 | 0.3 | 0.2 | 0.2 |
| 15-24 | 5.9 | 6.6 | 5.6 | 7.2 | 6.5 | 8.0 | 8.3 | 8.2 | 8.1 | 7.8 |
| 25-44 | 37.1 | 38.7 | 38.3 | 38.0 | 34.9 | 32.2 | 32.8 | 33.2 | 33.0 | 32.8 |
| 45-64 | 35.3 | 36.9 | 34.5 | 35.0 | 35.2 | 34.7 | 34.9 | 34.1 | 34.2 | 34.5 |
| 65-74 | 9.3 | 7.9 | 9.9 | 10.8 | 9.5 | 11.5 | 11.0 | 11.6 | 11.7 | 11.7 |
| 75+ | 11.9 | 9.9 | 11.4 | 8.7 | 13.7 | 13.5 | 12.7 | 12.6 | 12.8 | 13.1 |
| **Strong opioids** |  |  |  |  |  |  |  |  |  |  |
| Sex (% females) | 65.7 | 70.2 | 58.2 | 49.6 | 60.0 | 55.5 | 55.9 | 55.6 | 55.2 | 55.0 |
| Age category (%) |  |  |  |  |  |  |  |  |  |  |
| 0-14 | 0 | 0 | 0 | 0 | 0 | 0.1 | 0.1 | 0.1 | 0.1 | 0.1 |
| 15-24 | 0 | 0 | 0.9 | 0.9 | 0 | 2.9 | 3.3 | 3.2 | 3.3 | 3.3 |
| 25-44 | 10.8 | 9.1 | 14.5 | 11.5 | 7.0 | 18.6 | 19.5 | 20.5 | 20.9 | 20.6 |
| 45-64 | 17.1 | 17.3 | 18.2 | 15.0 | 13.5 | 29.9 | 30.5 | 30.4 | 30.5 | 30.6 |
| 65-74 | 14.4 | 5.0 | 10.9 | 13.3 | 15.2 | 15.6 | 15.2 | 15.1 | 15.0 | 15.3 |
| 75+ | 57.7 | 68.6 | 55.5 | 59.3 | 64.3 | 33.1 | 31.5 | 30.6 | 30.3 | 30.1 |
